# Historical availability of arable land affects contemporaneous female labor and health outcomes

**DOI:** 10.1371/journal.pone.0328083

**Published:** 2025-08-04

**Authors:** Chandan Kumar Jha, Sudipta Sarangi

**Affiliations:** 1 Madden College of Business & Economics, Le Moyne College, Syracuse, New York, United States of America; 2 Deptartment of Economics, Virginia Polytechnic Institute and State University, Blacksburg, Virginia, United States of America; Ariel University, ISRAEL

## Abstract

We contribute to the understanding of mechanisms underlying deep-rooted gender norms by exploring the link between the historical availability of arable land and contemporary gender outcomes. We argue that an abundance of arable land in historical times, *i.e.,* pre-industrial period, required more workers in the fields resulting in norms where women worked and contributed from outside the home as well. Consequently, these societies emphasized women’s health due to its positive effect on their productivity in the fields. Moreover, this economic contribution provided women greater bargaining power in the allocation of intrahousehold resources. The historical avail- ability of arable land for a nation is measured as the weighted mean of the shares of its constituent ethnic groups’ ancestral lands suited to cereal agriculture. Consistent with these arguments, we show that countries with more ancestral arable land have higher female labor force participation rates and better health outcomes, measured by maternal mortality ratio and female-male life expectancy gap. We then illustrate the ‘persistence of norm’ mechanism, by showing that ancestral arable land measured at the district level is positively associated with individual-level attitudes regarding women’s participation in the labor market.

## 1 Introduction

The World Economic Forum’s (WEF) 2022 Global Gender Gap Report [1] measures gender parity using four key dimensions: Economic Participation and Opportunity, Educational Attainment, Health and Survival, and Political Empowerment. The report argues that gender parity remains elusive: with the rate of progress we have seen in the last fifteen years, it will take 132 years to close the gender gap. It finds that there are significant differences in gender parity across countries, with Iceland having closed over 90 percent of the gender gap, and Afghanistan less than 50 percent. According to the latest figures (April 2025) made available by the World Bank [2] the proportion of national parliamentary seats held by women range from 0 percent in Oman, Tuvalu, and Yemen, to 63.8 percent in Rwanda, and female labor force participation ranges from less than 5 percent in Yemen to over 82 percent in Madagascar.

What explains these large variations in gender gaps in different socioeconomic dimensions across different countries? In recent years a number of different historical agricultural and ecological factors such as dietary practices [3], resource scarcity [4], the timing of the Neolithic Transition [5,6], and the adoption of the plough [7,8] have been shown to play a key role in shaping gender norms that continue to persist and affect women’s role and well-being in society even today. Yet, one fundamental component of agriculture that has not been examined by any of these studies is the *direct role* of the historical, *i.e.,* pre-industrial, availability of arable land even though it is a complementary input in nearly all the agro- ecological factors that have been used to explain modern gender inequality. Our paper explores this important aspect of the argument by examining how the historical availability of arable land, *i.e.,* ancestral land suited to cereal agriculture in the pre-industrial period (henceforth, referred to as the ancestral arable land), shaped gender norms regarding the appropriate role of women in society that continue to determine contemporary female labor force participation and women’s health outcomes.

We argue that the availability of ancestral arable land played a role in shaping norms regarding *female labor force participation*. Our argument proceeds as follows: an abundance of arable land would promote women’s participation in agricultural activities in the fields as opposed to just contributing from the confines of home simply because there would be a greater need for hands to work in the fields. This conjecture is supported by Boserup’s [9,10] thesis that the demand for labor in agriculture is affected by the type of production system. If the land is abundant relative to the labor, then every member of the family needs to contribute to agriculture, implying women also work outside the home (see for instance Fennell [11]). Consequently, societies with more arable land in historical (pre- industrial) times developed norms where women worked on agricultural lands and their economic contributions extended to agricultural output−a visibly measurable contribution unlike household duties.

Next, while existing studies only focus on norms regarding women’s appropriate role in society depending on whether women contributed to agriculture from inside the home or by working in agricultural fields, we recognize that historical factors could also influence norms regarding other gender outcomes that either influence or are influenced by the then appropriate role of women. To examine this, we distinguish between various gender outcomes: health, empowerment, and the labor market. We argue that societies with more ancestral arable land would develop norms where women’s health was considered important due to two important reasons. One, women’s economic contribution in these societies led to an increase in their bargaining power and hence a greater share in the intra-household allocation of resources, resulting in better health outcomes for them. It is well-documented that eco- nomic contributions increase women’s bargaining power in the allocation of intra-household resources and result in better well-being especially health outcomes [12,13]. Two, since women’s health was important for their productivity in agricultural fields, these societies developed norms where women’s health was considered more important than otherwise similar societies with a scarcity of arable land. Moreover, in those times, women’s empowerment, however, was not an important factor in their agricultural productivity. Hence, ancestral arable land would have no effect on norms regarding women’s empowerment, as measured by educational attainment and representation in national parliaments. These gender norms eventually became ingrained in the culture and even today we can expect to see higher female labor force participation and better female health indicators in these societies but not better female empowerment outcomes.

A second mechanism through which the lack of pre-industrial ancestral arable land in antiquity might have resulted in worse health outcomes for women is through resource scarcity. Multiple studies have suggested that resource scarcity contributed to gender inequality in history [4], prehistory [14], hunter-gatherer societies [15], and even among primates [16]. However, this has not been explored in the context of arable land in the pre-industrial period. In societies with a scarcity of arable land, there would also be fewer resources available for subsistence leading to more intense bargaining for the intra-household allocation of resources. Men would have an upper hand in this bargaining process because of their advantage over women in physical strength [7,17,18] and also due to existing gender norms that favored men [14,15]. Moreover, scholars have argued that “the male comparative advantage in brawn was accentuated by growing land scarcity, which increased the value not only of a man’s labor but also his ability to defend the farm against marauders” [17]. And, societies that were exposed to external threats or resource scarcity (among other ecological factors), are more likely to punish deviance and hence reinforce existing social norms such as gender norms [19]. Consequently, male-favoring norms would become even stronger in societies with a scarcity of arable land, and continue to affect women’s status in society by becoming a part of the culture.

To summarize, we hypothesize that the historical availability of arable land played a role

in shaping gender norms that promoted female labor force participation. Since women’s health was a factor in their productivity in agricultural fields, and since economic contributions increase women’s bargaining power in the allocation of intra-household resources, the abundance of arable land also led to better health outcomes for them. Eventually these behaviors became ingrained in the culture and continue to impact women’s labor force participation and their health outcomes even today. Consistent with our hypotheses, we show that ancestral arable land is positively associated with female labor force participation rates and female health outcomes across countries. Moreover, we show that residents of sub- national regions whose ancestors were endowed with more arable land are less likely to agree with the statement that men should have more right to a job than women when jobs are scarce. This is an important part of our analysis as it not only supports the ‘shaping gender norms’ conjecture but also rules out the possibility that our results could be driven by heterogeneity across countries.

Our paper contributes to the literature documenting the role of historical agricultural and ecological factors in shaping gender norms. Diamond [20] postulates that the adoption of agriculture may have contributed to both gross social and gender inequality. Hansen, Jensen, and Skovsgaard [5] show that the Neolithic timing of transition is negatively correlated with female labor force participation and other measures of gender inequality such as the share of women in parliament and female-male schooling ratio. Boserup’ 1970 seminal work argues that the adoption of the plough led to a gendered division of labor in which the role of women was principally domestic. Accordingly, Alesina, Giuliano, and Nunn [7] show that female labor force participation, female ownership of businesses, and female representation in parliament are lower in societies whose ancestors traditionally employed the plough. Uberti and Douarin [21] find that the cultural norms shaped by the use of the plough deter- mines the “Feminisation U” by showing that the U-shaped relationship between economic development and female labor force participation holds only for countries whose ancestors employed plough. Alesina, Giuliano, and Nunn [8] show that average male-to-female sex ratios are higher in societies whose ancestors traditionally employed the plough. Fredriksson and Gupta [6] find that male-to-female sex ratio is positively associated with longer history of agriculture, while Hazarika, Jha, and Sarangi [4] find that there are fewer missing women in the societies with greater average annual caloric yields per hectare based on the agro-climatic yields of pre-Columbian crops. In a recent study, Jha, Sarangi, and Tripathi [22] investigate the roles of these historical agro-ecological factors in diverse contexts including the labor force, politics, and business, and find them to be significantly associated with individual attitudes regarding women’s democratic and labor market rights as well as their abilities as political leaders and business executives. We refer interested readers to Jha, Sarangi, and Tripathi [22] for detailed review of literature on this topic.

## 2 Data and empirical strategy

### 2.1 Cross-country analysis

#### 2.1.1 Data and variables.

We utilize the ancestral arable land data provided by Alesina, Giuliano, and Nunn [7] as the measure of historical availability of arable land, that is, suitability of land to cereal agriculture in the pre-industrial period. These authors use the following steps to calculate nations’ ancestral arable land suited to cereal agriculture. First, the geographic coordinates of the centroid of each ethnic group are identified using records from George Peter Murdock’s Ethnographic Atlas. The land within a 200 kilometers radius of each centroid is considered as the ethnic group’s ancestral land. Second, the suitability of land for cultivating six major crops, that is, barley, wheat, rye, sorghum, foxtail millet, and pearl millet, is assessed using the FAO’s Global Agro-Ecological Zones (GAEZ) 2002 database. These crops are selected due to their similarity in numerous dimensions, including historical cultivation in the Eastern Hemisphere since the Neolithic Revolution, use for flour, porridge, bread, or beverages, and similar yields. The land’s suitability for cultivating these crops is assessed using factors like precipitation, temperature, soil slope, and soil characteristics. Third, the distribution of ethnic groups across the world today is estimated using the Ethnologue database, which is combined with population data from the Landscan 2000 database to link historical ethnicities to modern geographic locations. Finally, the share of a nation’s ancestral land suited cereal to agriculture is computed as the weighted mean of the shares of its constituent ethnic groups’ ancestral lands suited to cereal agriculture. As already noted, the weights are given by the shares of these different ethnic groups in the national population.

The data pertains to the pre-industrial period, capturing the characteristics of ethnic groups prior to industrialization. For some ethnic groups, the information dates back to the earliest written records or observations by early ethnographers, while for others, it is based on data from the twentieth century, reconstructed to reflect pre-industrial conditions. While the GAEZ data itself is modern, it models the suitability of land for crop cultivation based on geo-climatic characteristics that would have been relevant during the pre-industrial period. These characteristics include precipitation, temperature, soil slope, soil depth, and other environmental factors that are largely stable over time. The suitability measures are calculated for the land traditionally inhabited by ethnic groups prior to industrialization, making them reflective of pre-industrial agricultural conditions.

Our main dependent variables are female labor force participation rate and two indicators of women’s health: maternal mortality ratio and female-male life expectancy gap. We obtain the data for the female labor force participation rate and maternal mortality ratio from the 2013 Human Development Report [23]. The female labor force participation rate is defined as the percentage of females ages fifteen or older who are in the labor force. The maternal mortality ratio is computed as the number of maternal deaths per 100,000 live births. The data for female-male life expectancy gap is taken from the 2013 Human Development Report. Women’s empowerment indicators (female-male secondary education gap and the share of women in parliament) that we use for robustness checks also come from the 2013 Human Development Report [23]. Data for covariates come from various sources which are described as these variables are introduced in the paper.

#### 2.1.2 Empirical strategy.

We estimate the following linear regression equation using the ordinary least squares (OLS):


$$\begin{array}{c} y_{i}=\;\beta_{1}+\;\beta_{2}AAL_{i}+\gamma^{\prime }X_{i}+\;\varepsilon_{i} \end{array}$$ (1)

where the subscript *i* denotes country, the regressors $X_{i}$consist of a host of contemporaneous and historical covariates, and $\varepsilon_{i}$ represents regression error terms. $AAL_{i}$ is the fraction of ancestral land area suitable to the cultivation of six major crops mentioned above. We use $y_{i}$ to denote our dependent variables for country *i*, primarily, the female labor force participation rate, maternal mortality ratio, and the female-male life expectancy gap. Additionally, we use women’s empowerment indicators (female-male secondary education gap and the share of women in parliament) as dependent variables for falsification test.

In the baseline specification, we include 2012 per capita income in all our regression specifications to address concerns that the relationship between historical arable land and female labor force and health outcomes may have been driven by the omission of current resource environments that are likely to be correlated with both arable land and gender inequality at present. Data has been taken from the World Development Indicators. In addition to per capita income in 2012, our baseline specification also includes the following covariates. First, the fraction of land in the tropics is included as a predictor because “*tropical regions are hindered in development relative to temperate regions, probably because of higher disease burdens and limitations on agricultural productivity* [24].” This is important since pathogen prevalence has been found to shape culture regarding gender inequality [25]. Finally, the distance from the coast or sea-navigable river is included in our baseline specification since it was an important factor in trade and may have played a role in the exchange of culture including that related to gender inequality. Additionally, we include continent dummies in our baseline specification and in other specifications thereafter. The continent dummy is a dichotomous variable that takes the value 1 for countries in the relevant continent and zero otherwise. Summary statistics for these variables have been reported in Table 1. The correlation coefficients reported in Table S1 in S1 Supplementary Material show that the correlation among the baseline variables is not substantial.

**Table 1:** Summary statistics.

Variable	Mean	Std. Dev.	N
Female labor force participation (%)	52.947	16.174	134
Maternal mortality ratio	164.358	211.945	134
Female-male life expectancy gap at birth	4.869	2.532	134
Share of women in national parliaments (%)	19.945	10.784	134
Ancestral arable land	0.54	0.321	134
Migration-adjusted potential arable land	0.464	0.244	133
Potential arable land (migration-unadjusted)	0.435	0.271	133
Log(Per capita income)	8.605	1.538	134
Land area in the geographical tropics	0.455	0.476	134
Mean distance to nearest coastline or sea-navigable river (in ’000kms)	0.33	0.448	134
Years since Neolithic Transition (migration-adjusted)	5.44	2.063	133
Fraction of population with ancestors who used the plough	0.572	0.467	134
Pre-1500 CE average crop yield (ancestry-adjusted)	1386.1	692.57	133
Index of democracy in 2000	3.862	6.389	130
State Antiquity Index	0.457	0.242	122
Origins of national legal system = Britain	0.291	0.456	134
Origins of national legal system = France	0.552	0.499	134
Origins of national legal system = Germany	0.127	0.334	134
Origins of national legal system = Scandinavia	0.03	0.171	134
Religious Fractionalization	0.430	0.235	134
Percentage share of agriculture in GDP	0.125	0.127	134
Percentage share of industry in GDP	0.311	0.128	134

The female labor force participation rate is defined as the percentage of females ages fifteen or older who are in the labor force. The maternal mortality ratio is computed as the number of maternal deaths per 100,000 live births. Both migration-adjusted potential arable land and ancestral arable land are the fractions of total land area in the respective categories. Religious fractionalization, taken from Alesina et al. [26], reflects the likelihood that two randomly selected individuals from a given population will belong to different religious groups. The State Antiquity Index, taken from Bockstette, Chanda, and Putterman [27], is a measure of how long a given territory has experienced state-level political organization. The data source for shares of agriculture and services in GDP is the CIA Factbook.

### 2.2 Subnational analysis

#### 2.2.1 Data and variables.

We rely on the publicly available data from Alesina, Giuliano, and Nunn [7] for this analysis. We utilize data from three waves of the WVS covering the period 1995–2007 to explore the link between ancestral arable land in the different parts of a country and individuals’ attitudes about women’s labor market participation captured by the following question

“When jobs are scarce, men should have more right to a job than women”

This variable reflects differences in cultural beliefs regarding women’s right to a job relative to men’s. Individuals responses are coded as ‘disagree’ (0) and ‘agree’ (1). Our hypothesis suggests that, on average, more responses would be recorded as “agree” in countries with more ancestral arable land to reflect the norms that these societies developed where women working outside the fields was acceptable. Clearly, these beliefs might play a role in women’s labor force participation, especially, when times are tough and jobs are scarce. Moreover, since our hypotheses are based on the persistence of gender norms, a significant association between individual perceptions reflecting gender norms corroborates our mechanism.

We utilize the measure of ancestral availability of arable land at the sub-national level from Alesina, Giuliano, and Nunn [7]. Note that the analysis in this section is limited by our

Inability to modify the publicly available data from Alesina, Giuliano, and Nunn [7] because the data does not identify the subnational regions. The available data, however, allows us to examine the association between ancestral arable land and individual attitudes reflecting norms regarding women’s labor market participation. The data was accessed from the following web-page (available as of July, 24, 2025): https://scholar.harvard.edu/files/nunn/files/alesina_giuliano_nunn_qje_2013_replication_materials.zip.

#### 2.2.2 Empirical strategy.

We estimate the following equation using OLS and logit models:


$$\begin{array}{c} y_{i,d,c}=\alpha_{c}+\beta AAL_{d}+\;Xi\prime \theta +\;Xd^{\prime }H\delta +\;\varepsilon_{i,d,}\; \end{array}$$ (2)

where *i*, *d*, and *c* denote an individual, a district, and a country, respectively. Here *α*_*c*_ denotes country-fixed effects and $X_{i}$ denotes individual-level characteristics. $AAL_{d}$ is our primary variable of interest and is measured at the district-level. The $X_{d}^{H}$ denotes historical district factors and includes ancestral plough use, fraction of ancestral land that was tropical or subtropical, ancestral domestication of large animals, ancestral settlement patterns, and ancestral political complexity. Individual-level variables include age, age-squared, gender, marital status, and dummies for primary and secondary education. Country-level variables include income per capita and income per capita squared (both in natural logs), measured in the same year as the dependent variable.

## 3 Results

### 3.1 Cross-country results

#### 3.1.1 Female labor force participation.

We start by showing the association between ancestral arable land and contemporary female labor force participation rates in Table 2. Column 1 presents the estimates of the empirical specification without any covariates except for the continent dummies. The coefficient of ancestral arable land is statistically highly significant and positive, indicating that countries

**Table 2:** Ancestral Arable Land and Female Labor Force Participation.

	(1)	(2)	(3)	(4)	(5)	(6)
Ancestral arable land	13.40^***^	18.22^***^	18.18^***^	12.40^**^	9.449^**^	12.02^**^
	(4.712)	(5.508)	(5.351)	(4.899)	(4.734)	(5.221)
Log(per capita income)		−21.84^**^	−21.86^***^	−21.37^***^	−22.90^***^	−14.54
		(8.354)	(8.349)	(7.649)	(7.499)	(9.477)
ln(Per capita income)		1.233^***^	1.234^***^	1.196^***^	1.347^***^	0.971^*^
squared		(0.438)	(0.439)	(0.412)	(0.412)	(0.513)
Land area in the		13.70^***^	12.15^**^	4.946	8.551^*^	12.84^**^
geographical tropics		(5.221)	(5.774)	(5.341)	(5.112)	(6.224)
Distance from nearest		3.502	2.855	2.176	6.399^***^	6.189^***^
coastline/river		(2.485)	(2.639)	(2.448)	(2.210)	(2.291)
Ancestral plough use			−4.084	−4.847	−3.041	0.0667
			(6.187)	(6.180)	(5.125)	(4.657)
Years since Neolithic Transition				−4.397^***^ (0.921)	−4.466^***^ (0.974)	−3.844^***^ (1.157)
Pre-1500 CE crop yield					0.00722^***^ (0.00213)	0.0073^***^ (0.00235)
Additional controls Continent Dummies	Yes	Yes	Yes	Yes	Yes	Yes Yes
Observations	134	134	134	133	133	122
Adjusted *R*^2^	0.302	0.384	0.382	0.489	0.536	0.531

The dependent variable is female labor force participation. Each column presents the results of a different multivariate regression specification. The significance of each explanatory variable is assessed using a *t*−test. OLS coefficients are reported with robust standard errors in parentheses. ^*^ **p <* *0*.*10, ^**^ *p < *0*.*05,

*** *p <*0*.*01. Constant not reported. Additional controls: democracy index, legal origin dummies, state antiquity index, religious fractionalization, shares of agriculture and industry in GDP.

with more ancestral arable land have higher levels of female labor force participation rates at present. Column 2 presents the results of our baseline specification given in equation1 along with the continent dummies. The coefficient estimates become even larger upon the inclusion of these covariates and remain highly statistically significant. Note that the association between ancestral arable land and female labor force participation remains unchanged when we do not include GDP per capita and its squared term as covariates (Tables S4 and S5 in S1 Supplementary Material).

Could it be possible that ancestral arable land is simply capturing the association between ancestral arable land and plough positive crops? As reported in Table S2 in S1 Supplementary Material, the correlation coefficient between ancestral arable land and plough-positive crops in our sample is small (*ρ *= 0*.*36), mitigating concerns that the observed association between ancestral arable land and female labor force participation rates could be capturing the effects of plough positive crops on women’s participation in the labor force. This concern is further reduced when the association between ancestral arable land and female labor force participation remains un- changed when we include ancestral plough use as an additional explanatory variable, which is highly correlated with plough positive crops (*ρ *= 0*.*77), in column 3. In addition, our results remain unchanged when plough positive and negative crops are included as explanatory variables (results available from authors on request). Our results also remain unchanged when we account for the presence of large animals and ancestral dependence on animal husbandry for subsistence in our model using the data provided on this by Alesina, Giuliano, and Nunn [7]. These results are reported in Table S6 in S1 Supplementary Material.

The positive association between ancestral arable land and female labor force participation rates remains statistically significant at conventional levels in the next columns when we include other historical factors (besides the ancestral use of the plough [7,8]) that have been shown to influence gender inequality: the (ancestry-adjusted) number of years since a country has moved to agriculture in column 4 [5,6], and the ancestry-adjusted pre-1500 CE crop yield in column 5 [4]. Although the inclusion of the last two variables causes the coefficient of ancestral arable land to drop, it still remains economically and statistically significant.

Additionally, we include a number of covariates to account for economic, institutional, and cultural heterogeneity across countries. We use the widely-used Polity2 Index (http://www.systemicpeace.org/polityproject.html) for the year 2000 as a measure of democracy. The index takes values in the range of −10 (hereditary monarchy) to +10 (consolidated democracy). It has been argued that all national legal systems are of either British, French, German, or Scandinavian extraction [28]. Since the laws of Britain, France, Germany, and Scandinavia differ in their support of private market outcomes, the origins of nations’ legal systems may be a significant influence upon their economies, impacting the current levels of present gender inequality and female labor force participation. Hence, we account for the legal origins of nations. In our sample, approximately 55 percent nations have French legal system origins, about 29 and 13 percent countries have British and German legal origins. We include religious fractionalization index (using data from Alesina et al. [26]) as an explanatory variable to account for cultural heterogeneity across countries. Religious fractionalization reflects the probability that two randomly selected individuals from a given population will belong to different religious groups. Further, a country’s history of the level of sophistication of political organization within its borders is accounted for using a measured of the State Antiquity Index [27]. The State Antiquity Index is a measure of how long a given territory has experienced state-level political organization, and quantifies the depth of a country’s historical experience with state institutions. We account for the shares of agriculture and industry in GDP because countries that are predominantly agricultural may have greater female labor force participation rates. As can be seen in the last column of Table 2, the positive relationship between ancestral arable land and female labor force participation rates remains robust to the inclusion of the above variables. These results remain unchanged if we introduce these variables one-by-one (see Table S7 in S1 Supplementary Material).

The lowest coefficient estimate reported in column 5 of Table 2 indicates that a one standard deviation increase (0.32 on a scale of 0–1) in the ancestral arable land is associated with 3 percentage points increase in the female labor force participation rate. Therefore, a country with one standard deviation higher ancestral arable land would have 3 percentage points more women in the labor force, representing a 5.6% increase relative to the mean labor force participation rate, compared to an otherwise similar country.

#### 3.1.2 Women’s health.

Earlier, we argued that the pre-industrial availability of arable land led to better outcomes for women due to their direct contribution to agricultural fields. This direct contribution not only required them to be healthy but also gave them greater bargaining power in the allocation of intra-household resources. We empirically examine this hypothesis and present the results in Table 3 with women’s health indicators, maternal mortality ratio and female- male life expectancy gap, being the dependent variables.

**Table 3:** Ancestral Arable Land and Women’s Health.

	Maternal Mortality Ratio				Female-Male Life Expectancy Gap			
	(1)	(2)	(3)	(4)	(5)	(6)	(7)	(8)
Ancestral arable land	−115.7^**^	−139.7^***^	−133.5^***^	−108.2^**^	1.308^*^	2.075^***^	1.467^**^	1.757^**^
	(48.10)	(44.83)	(46.57)	(54.47)	(0.681)	(0.569)	(0.636)	(0.710)
Ancestral plough use			48.02^*^	65.38^**^			−0.728	−0.458
			(27.88)	(31.84)			(0.631)	(0.673)
Years since			−1.424	−4.027			−0.193	−0.209
Neolithic Transition			(6.274)	(7.254)			(0.133)	(0.145)
Pre-1500 CE crop yield			−0.0172	−0.0181			0.0009^***^	0.0005^*^
			(0.0126)	(0.0156)			(0.0003)	(0.0003)
Baseline controls	Yes		Yes	Yes	Yes		Yes	Yes
Additional controls				Yes				Yes
Continent dummies	Yes	Yes	Yes	Yes	Yes	Yes	Yes	Yes
Observations	134	134	133	122	134	134	133	122
Adjusted *R*^2^	0.689	0.795	0.795	0.803	0.364	0.587	0.623	0.665

The dependent variable is maternal mortality ratio in columns 1–4 and female-male life expectancy gap in columns 5–8. Each column presents the results of a different multivariate regression specification. The significance of each explanatory variable is assessed using a *t*−test. OLS coefficients are reported with robust standard errors in parentheses. ^*^ **p <* *0*.*10, ^**^ **p <* *0*.*05, ^***^ *p < *0*.*01. Baseline controls: Log(per capita income) and its squared term, land area in geographical tropics, distance to nearest coastline or sea-navigable river. Additional controls: democracy index, legal origin dummies, state antiquity index, religious fractionalization, shares of agriculture and industry in GDP. Constant not reported.

To conserve space, for health indicators, we present the results of four different specifications by cumulatively adding additional covariates: the first without any covariate, the second with baseline explanatory variables, the third with historical agro-ecological factors, and the final specification includes various economic, institutions, and cultural factors as in the last column of Table 2. However, the results remain robust when we introduce additional covariates one-by-one as in Table 2. The coefficient estimates of ancestral arable land are negative and statistically significant in the first four columns of Table 3, indicating a negative association between ancestral arable land and maternal mortality ratio. As per the lowest coefficient in column 4, all else equal, a country with one standard deviation more ancestral arable land would experience approximately 35 fewer maternal deaths per 100,000 population. With the mean value of maternal deaths in our sample being 164 in 100,000, this effect is sizable representing over 21% decline in maternal deaths relative to the mean. Similarly, the association between ancestral arable land and female-male life expectancy, as expected, is positive and statistically significant at conventional levels in the last three columns. As per the lowest coefficient estimate reported in column 5, a one standard deviation increase in ancestral arable land is associated with an increase in female-male life expectancy gap by about 0.42 years, representing about 8.6% increase relative to the mean female-male life expectancy gap of 4.87 years.

Since one could argue that women’s participation in the labor force could influence their health and empowerment indicators, the association between ancestral arable land and women’s health and empowerment indicators could be driven by the omission of norms regarding female labor force participation. Hence, it is important to note that women’s participation in the labor force is not highly correlated with any of the health and empowerment indicators as shown in Table S3 in S1 Supplementary Material: maternal mortality ratio, female-male life expectancy gap, women’s share in national parliaments, and female-male secondary education gap. Moreover, the association between ancestral arable land and women’s health indicators barely changes when current female male participation rates are included in the model as shown in Table S8 in S1 Supplementary Material. The coefficient estimates of ancestral arable land are statistically significant at conventional levels in all columns except for column 8 in which it is barely insignificant (**p* *= 0*.*113).

We further argue that since women’s empowerment was not a factor for their agricultural contribution in the pre-industrial period, we should not expect a significant association between ancestral arable land and women’s empowerment indicators. Results reported in Table 4 support this conjecture as the coefficient of ancestral arable is statistically insignificant in every specification reported in columns 1–8. We do not find evidence of significant positive connections between the availability of arable land in the pre-industrial period and female-male secondary education gap (columns 1–4) and ancestral arable land and women’s share in national parliaments (columns 5–8). It is worth noting that this exercise also serves as a falsification test. A falsification test is used to evaluate the validity of a hypothesis by attempting to demonstrate that it is false. Suppose that the significant association between ancestral arable land and women’s health indicators was driven by some unobserved omitted factors favorable to women. Then, we should have observed a positive association between ancestral arable land and women’s empowerment indicators as well. The absence of a significant association between ancestral arable land and women’s empowerment indicators falsifies the hypothesis that such an omitted factor is creating a spurious relationship between ancestral arable land and female labor and health outcomes.

**Table 4:** Falsification Test: Ancestral Arable Land and Women Empowerment.

	Female-Male Secondary Education Gap				Share of Women inParliament			
	(1)	(2)	(3)	(4)	(5)	(6)	(7)	(8)
Ancestral arable land	−0.916	−0.592	−2.601	−3.771	4.594	2.207	0.803	−1.160
	(3.359)	(3.263)	(3.349)	(3.713)	(3.508)	(4.001)	(4.244)	(5.175)
Ancestral plough use			−0.147	0.218			−2.090	−2.388
			(2.301)	(2.365)			(3.543)	(3.666)
Years since			−1.587^***^	−1.262^**^			−1.161^*^	−0.915
Neolithic Transition			(0.523)	(0.531)			(0.636)	(0.810)
Pre-1500 CE crop yield			−0.0004 (0.0013)	−0.0001 (0.0014)			−0.0006 (0.0019)	0.00022 (0.0023)
Baseline controls	Yes		Yes	Yes	Yes		Yes	Yes
Additional controls Continent dummies	Yes	Yes	Yes	Yes Yes	Yes	Yes	Yes	Yes Yes
Observations	134	134	133	122	134	134	133	122
Adjusted *R*^2^	0.084	0.245	0.288	0.313	0.085	0.093	0.089	0.123

The dependent variable is female-male secondary education gap in columns 1–4 and the share of women in national parliaments in columns 5–8. Each column presents the results of a different multivariate regression specification. The significance of each explanatory variable is assessed using a *t*−test. OLS coefficients are reported with robust standard errors in parentheses. ^*^ **p <* *0*.*10, ^**^ **p <* *0*.*05, ^***^ *p < *0*.*01. Baseline controls: Log(per capita income) and its squared term, land area in geographical tropics, distance to nearest coastline or sea-navigable river. Additional controls: democracy index, legal origin dummies, state antiquity index, religious fractionalization, shares of agriculture and industry in GDP. Constant not reported.

### 3.2 Subnational results

We have argued that the availability of arable land in history shaped cultural norms regarding women’s labor force participation and these norms have been transmitted through generations. In this section, we provide evidence of a significant connection between ancestral arable land and individual perceptions reflecting the norms regarding female labor force participation. Using individual-level data from the World Values Survey (WVS), we examine whether ancestral arable land, measured at the district-level, can explain respondents’ perception regarding women’s labor market participation. Besides the fact that this analysis corroborates our cultural persistence mechanism, it has two important advantages over the cross-country analyses that we have relied on so far. First, while the female labor force participation rate, maternal mortality ratio, and female-male life expectancy used in the cross-country analyses can be considered as objective measures of gender inequality, WVS responses are subjective measures that reflect norms and values of the respondents. Second, individual-level data with over 80,000 observations from at least 70 countries across the world allows us to look at this relationship after accounting for individual and district level variables along with country dummies. This rules out the possibility that these results are driven due to the omission of country-specific fixed factors.

The results are reported in Table 5. We report the coefficient estimates obtained from various specifications estimated using the OLS in Panel A. Since the dependent variable is a binary variable, we also estimate these models using logistic regressions and report the marginal effects of our variable of interest, ancestral arable land, in Panel B. We begin by estimating the model with only individual-level characteristics as explanatory variables in column 1 and subsequently add district-level variables in the next columns. In columns 1–3, we include country dummies to rule out the possibility that these results could be driven due to the omission of country-specific fixed factors. Finally, the last column reports results of the specification that includes country characteristics along with continent dummies as in the specification reported in column 3 of Table V of Alesina, Giuliano, and Nunn [7]. Note, however, that they report the coefficient of ancestral plough use while our variable of interest is ancestral arable land.

**Table 5:** Ancestral Arable Land and Norms Regarding Women’s Labor Market Rights.

	(1)	(2)	(3)	(4)
OLS Estimates				
Ancestral arable land	-0.451^***^	−0.241^***^	−0.196^***^	−0.177^***^
	(0.00492)	(0.0111)	(0.0642)	(0.0444)
Individual-level controls	Yes	Yes	Yes	Yes
District-level controls		Yes	Yes	Yes
Contemporary country controls				Yes
Fixed Effects	Country	Country	Country	Continent
Countries	74	74	74	70
Districts	728	728	700	674
Observations	89766	89766	87528	80303
Adjusted *R*^2^	0.135	0.274	0.275	0.206
	Marginal Effects from Logit Estimation			
Ancestral arable land	-0.502***(0.00597)	−0.285*** (0.0144)	−0.263^***^ (0.0818)	−0.221*** (0.0574)

Panel A reports OLS coefficient estimates; standard errors clustered at the district-level in parentheses. Panel B reports the marginal effects computed at the means of the covariates of the same specifications in Panel A estimated using the OLS; delta-method standard errors in parentheses. * **p <* *0*.*10, ** **p <* *0*.*05, *** **p <* *0*.*01. The dependent variable is responses (“yes” coded as 1 and “no” coded as 0) to the following WVS question: “When jobs are scarce, men should have more right to a job than women”. Individual-level controls: age, age^2^, dummies for primary and secondary education, gender, and dummy for being married. District-level controls: ancestral plough use, fraction of ancestral land that was tropical or subtropical, ancestral domestication of large animals, ancestral settlement patterns, and ancestral political complexity. Country-level controls: income per capita and income per capita squared in natural logs measured in the same year, as the dependent variable.

The coefficient of ancestral arable land is negative and statistically highly significant in all the columns of Table 5. These estimates show that residents of districts with ancestral lands better suited to agriculture are significantly less likely to agree with the statement that men ought to have more right to a scarce job than women. The marginal effects, computed at the mean of the covariates, obtained from the logistics regressions are negative and significant, suggesting that the availability of ancestral arable land is positively associated with individual perceptions regarding women’s labor market participation. These results thus support our hypothesis that the availability of arable land in the pre-industrial period played a role in shaping norms regarding women’s labor market participation and thereby continues to affect female labor force participation in modern times.

## 4 Robustness checks

While we include GDP per capita in our empirical analysis to account for contemporary economic development, there might still be some concerns that our measure of the historic availability of arable land, *i.e.,* ancestral arable land may not pertain to historical times, but rather may be capturing a contemporaneous effect. To address this concern, we carry out several robustness checks. The findings of these robustness checks indicate that this is not the case.

### 4.1 An alternative measure of historical arable land

First, we check the robustness of our estimates using an alternative measure of the historical suitability of land for cultivation. The Food and Agricultural Organization (FAO) provides estimates of each country’s potential arable land [29]. In most cases, potential arable land exceeds actual arable land, in that a portion of potential arable land, such as currently forested land, has not yet been brought under cultivation. In a few countries, like Egypt however, modern irrigation has permitted actual arable land to exceed land suited to rainfed cultivation.

The FAO bases its estimates of potential arable land on a soil map of the world that identifies major soil constraints such as salinity, a global climatic database, and a database of the climatic and soil requirements of 21 major crops. These crops are maize, wheat, rice, sugar cane, soybeans, potatoes, onions, tomatoes, beans, peas, lentils, chickpeas, sorghum, barley, rye, millet, cassava, sugar beet, cotton, oil palm, and groundnuts. The FAO’s estimate of a country’s current potential arable land is a plausible measure of its historical resource endowment for the following reasons. First, agriculture has been the mainstay of mankind since the Neolithic Revolution 12,000 years ago, and potential arable land speaks to the agricultural potential of a region in the absence of modern irrigation and technologies that mitigate soil constraints. Second, a modern soil map of the world is also historical, as are the climatic and soil requirements of mankind’s main crops, in that almost nothing has changed in their regard. Third, while the world’s climate has seen considerable change during the geological epoch of the Holocene, within which the Neolithic Revolution occurred, it has, at any rate, been fairly stable for the past one to two millennia [30].

A noteworthy point here is that while FAO’s estimate of potential arable land is based on 21 major crops, the ancestral arable land computed by Alesina, Giuliano, and Nunn [7] is based on only 6 major crops. We adjust the current potential arable land to account for the migration since 1500 CE using the Putterman-Weil migration index [31] as evidence suggests that exposure to a different gender norm causes a change in a population’s gender norms and attitudes through cultural transmission [32]. The migration-adjusted potential arable land of a country is computed as the weighted average of the potential arable land of all the countries from where its population originated with the weight being the proportion of the country’s long-term residents belonging to the source country in 2000. As shown in Table S2 in S1 Supplementary Material, the correlation between the two measures for our sample, though statistically significant, is not very high (*ρ *= 0*.*44) suggesting that the use of this measure is useful for robustness check.

Results presented in Table 6 show that migration-adjusted potential arable land is positively and significantly associated with female labor force participation rates in columns 1–5 when various historical and contemporaneous covariates are added to the model. As can be seen in Table S2 in S1 Supplementary Material, there is a high correlation between migration-adjusted potential arable land and pre-1500 CE crop yield (*ρ *= 0*.*52, **p <* *0*.*00) in our sample because historical availability of land was an important factor determining the yield in those times. Thus, the inclusion of ancestry-adjusted pre-1500 CE crop yield in column 4 might be capturing some of the effects of migration-adjusted current potential arable land causing its coefficient to drop significantly in column 4. In column 5, when we account for several economic and institutional factors along with ancestry-adjusted pre-1500 CE crop yield, the coefficient of migration-adjusted potential arable land loses statistical significance at the conventional levels (**p <* *0*.*11). However, when this variable is dropped from the model, the association be- tween migration-adjusted current potential arable land and female labor force participation turns out not only statistically significant at the conventional levels but is also significantly bigger in the last column.

**Table 6:** Migration-Adjusted Current Potential Arable Land and Female Labor Force Participation.

	(1)	(2)	(3)	(4)	(5)	(6)
Migration-adjusted	20.07^***^	19.84^***^	17.95^***^	10.96^*^	9.476	14.95^**^
potential arable land	(7.539)	(7.452)	(6.373)	(6.174)	(5.850)	(5.907)
Ancestral plough use		−3.478	−4.242	−2.971	−0.625	−1.805
		(5.411)	(5.351)	(4.843)	(4.475)	(4.749)
Years since			−4.756^***^	−4.773^***^	−4.221^***^	−4.146^***^
Neolithic Transition			(0.894)	(0.919)	(1.130)	(1.093)
Pre-1500 CE crop yield				0.0062^***^ (0.0022)	0.0063 (0.0025)	
Baseline controls	Yes	Yes	Yes	Yes	Yes	Yes
Additional controls			Yes	Yes	Yes	Yes
Continent dummies	Yes	Yes			Yes	Yes
Observations	133	133	133	133	122	122
Adjusted *R*^2^	0.376	0.373	0.506	0.533	0.518	0.494

The dependent variable is female labor force participation rate. Each column presents the results of a different multivariate regression specification. The significance of each explanatory variable is assessed using a *t*−test. OLS coefficients are reported with robust standard errors in parentheses. ^*^ **p <* *0*.*10, ^**^ **p <* *0*.*05, ^***^ *p < *0*.*01. Baseline controls: Log(per capita income) and its squared term, land area in geographical tropics, distance to nearest coastline or sea-navigable river. Additional controls: democracy index, legal origin dummies, state antiquity index, religious fractionalization, shares of agriculture and industry in GDP. Constant not reported.

Additionally, we check the validity of the historicity of our arable land measure. Results presented in Table S10 in S1 Supplementary Material confirm the validity of our measure and are consistent with our claim that the observed effects are due to the transmission of norms.

### 4.2 Ancestral vs. current arable land: the role of norms

Next, we report the results of a horse race between ancestral arable land and current potential arable land. A horse race refers to a comparison of different models to see which performs best at explaining a particular phenomenon or making predictions. In our case, the horse race between these two land variables involves running regressions with both these variables in an empirical specification to determine which one is a stronger predictor of contemporary gender labor and health outcomes. The objective is to demonstrate that the observed effects of ancestral arable land on gender inequality are driven by the historical availability of arable land, and not by the omission of the availability of current potential arable land.

Results reported in Table 7 suggest that ancestral arable land has significant predictive power in explaining female labor force participation. The first three columns report the results with ancestral arable land, current potentially arable land, and both without any covariates (except for continent dummies), respectively. These unconditional regression estimates show both, ancestral and current arable land, to be significantly, positively associated with female labor force participation across countries. When the baseline specification is estimated without ancestral arable land and current potential arable land in column 4, the adjusted-*R*^2^ is 0*.*322. The adjusted-*R*^2^ rises to 0*.*384 following the inclusion of ancestral arable land in the model in column 6. When both ancestral arable land and current potential arable land are included in the model, then the adjusted-*R*^2^ further rises to 0*.*405. Further, both ancestral and current arable land appear with statistically significant sign in column 7, suggesting that both are significant predictors of female labor force participation. This result is intuitively appealing: While ancestral arable land would impact current female labor force participation through its effect on norms regarding women working outside the home, current arable land would encourage female labor force participation due to a greater need for labor in agricultural activity.

**Table 7:** Female Labor Force Participation: A Horse Race between Ancestral vs. Current Arable Land.

	(1)	(2)	(3)	(4)	(5)	(6)	(7)	(8)
Ancestral arable land	13.40^***^(4.712)		10.40^**^(4.462)			18.22^***^(5.508)	13.21^***^ (5.015)	9.974^*^ (5.277)
Current potential arable land		18.30^***^ (5.982)	15.77^***^ (5.895)		20.12^***^ (6.697)		14.09^**^ (6.348)	6.551 (5.609)
Baseline Controls		Yes			Yes	Yes	Yes	Yes
Additional Controls								Yes
Continent dummies	Yes	Yes	Yes	Yes	Yes	Yes	Yes	Yes
Observations	134	133	133	134	133	134	133	121
Adjusted *R*^2^	0.302	0.323	0.343	0.322	0.380	0.384	0.405	0.530

The dependent variable is female labor force participation rate. Each column presents the results of a different multivariate regression specification. The significance of each explanatory variable is assessed using a *t*−test. OLS coefficients are reported with robust standard errors in parentheses. ^*^ **p <* *0*.*10, ^**^ **p <* *0*.*05, ^***^ *p < *0*.*01. Baseline controls: Log(per capita income) and its squared term, land area in geographical tropics, distance to nearest coastline or sea-navigable river. Additional controls: ancestral plough use, pre-1500 CE crop yield, years since Neolithic Transition, democracy index, legal origins, religious fractionalization index, state antiquity index, shares of agriculture and industry in GDP. Constant not reported.

The relationship between ancestral arable land and female labor force participation remains robust when we include additional explanatory variables along with current potential arable land in the last column. Interestingly, we find that when additional covariates are added to the model, the availability of current potential arable land loses statistical significance at the conventional levels while the ancestral arable land remains significantly associated with female labor force participation. These results suggest that the norms shaped by the historical availability of arable land play a more important role in determining women’s participation in the labor force today than the availability of current arable land. Results are similar for female health outcomes in Table S11 in S1 Supplementary Material.

## 5 Discussion and conclusion

This paper contributes to our understanding of how events in the past affect current gender inequality in several important ways. We introduce a very important missing piece in the context of agricultural and ecological factors, namely, the availability of arable land in pre- industrial societies to study its role in shaping norms regarding appropriate gender roles, and demonstrate how it continues to exert an influence on modern gender outcomes. While earlier studies focus either only on women’s roles [5,7] or well-being, [4,6,8] our analysis connects the two and shows that ancestral arable land can influence gender well-being that is directly tied to its effects on gender roles. We show that ancestral arable land is positively associated with women’s labor force participation as well as women’s health indicators as health was important for women to be productive in the agricultural fields. Notably, the association between ancestral arable land remains a significant predictor of women’s labor force participation rates and their health outcomes despite accounting for the availability of current arable land, underscoring the role of cultural norms. Finally, we provide evidence of a significant association between the historical availability of arable land and individual perceptions reflecting norms regarding women’s labor force participation. Using the World

Values Survey (WVS) data, we show that residents of sub-national regions whose ancestors were endowed with more arable land are less likely to agree with the statement that men should have more right to a job than women when jobs are scarce.

With several historical factors having been identified by recent studies to influence con- temporary gender outcomes, we also assess their relative importance in affecting gender outcomes. To do so, we run regressions that include the historical agro-ecological factors identified by the recent literature [4,5,7] along with our variable of interest, ancestral arable land. We find that ancestral arable land is a significant correlate of all three variables–female labor force participation, maternal mortality ratio, and female-male life expectancy gap, and insignificant correlate of female-male secondary education gap and the share of women in parliament, which is consistent with our hypotheses. On the other hand, the transition to agriculture is significantly, negatively associated with female labor force participation and female-male secondary education gap, which is consistent with the findings of Hansen, Jensen, and Skovsgaard [5]. Interestingly, when all these historical agro-ecological variables are pitted against one another in the same empirical model, the ancestral plough use is significantly associated with only female-male life expectancy gap, with women being at a disadvantage. Finally, historical agricultural yield is positively associated with female labor force participation rate and negatively correlated with female-male secondary education gap. Results (presented in Table S9 in S1 Supplementary Material), thus, indicate that among all historical factors considered, ancestral arable land continues to be the most significant correlate of female labor force participation and women’s health outcomes.

The United Nation’s Sustainable Development Goals note that gender equality is a fundamental human right that must be pursued to ensure a peaceful, prosperous and sustainable world. Although gender equality is generally believed to be a by-product of economic development, the bidirectional causal association between economic development and women empowerment is too weak to be self-sustaining, requiring policy interventions to achieve gender parity [33]. Evidence shows that gender attitudes are passed on from parents to children [34,35], and social and cultural norms are important determinants of women’s labor force participation [36–38]. Such norms do not only prevent women from participating in certain jobs due to attached stigma [39] but also influence inequality in gender status as the persistence of gender stereotypes prevents women from demonstrating their competence in various economic and political arenas [40]. In the same vein, we show that the relationship between ancestral arable land and modern gender labor force and health outcomes remains significant despite accounting for measures of contemporary economic development, signifying the impact of norms created by economic conditions in the distant past.

Our results, along with the findings of many recent studies, therefore, emphasize that to bring about equality between men and women, it is important to uncover the mechanisms that led to gender norms determining present gender inequality, which can help design policies that work around a norm. Recent research can provide guidance in designing such policies. For instance, affirmative policy actions providing women access to public offices may be helpful in modifying attitudes regarding women’s capabilities as they weaken gender stereotypes [41] and, as discussed in Heath and Jayachandran [12], policies that generate employment opportunities (especially for women) can improve women’s bargaining power. Other studies find that conditional cash transfers that incentivize parents to invest in girls can improve sex ratio at birth and post-birth outcomes including immunization and education [42]. Importantly, since inter-generational transmission of preferences plays a role in gender differences in occupation, such policies need to be in place over the long-run as the society attempts to preserve its cultural values regarding women’s role in the society. On the other hand, policies that do not appropriately account for gender norms might fail to have similar outcomes for both genders as shown in a recent study by Wang and Zhang [43] who find that while a large-scale nutrition assistance program led to an increase in boys’ weight reducing their likelihood of being underweight, it had no effect on girls of similar age.

## Supporting information

S1 Supplementary Material Supplementary Material for historical availability of arable land affects contemporaneous female labor and health outcomes. The Supplementary Material provides additional tables showing the robustness of the results derived in the paper.
(PDF)

S1 File Replication package. This zip file contains the underlying dataset and the STATA do-file used to replicate the results of the manuscript.
(ZIP)
