# Supplementary material for: Historical availability of arable land affects contemporaneous female labor and health outcomes
Source: PLoS One. 2025 Aug 4;20(8):e0328083. doi: 10.1371/journal.pone.0328083 (PMC12321104; doi:10.1371/journal.pone.0328083)
Supplement: S1 Supplementary Material — The Supplementary Material provides additional tables showing the robustness of the results derived in the paper. (PDF) [file pone.0328083.s001.pdf]

S Supplementary Materials

Historical availability of arable land affects contemporaneous female labor and health outcomes

by

Chandan Kumar Jha and Sudipta Sarangi

Description of Tables

Tables S1, S2, and S3 provide the correlations among baseline covariates, historical factors, and gender outcomes used in this study, respectively.

Tables S4 and S5 show the robustness of association between ancestral arable land and female labor force participation rate without GDP per capita included in the regression specifications. Table S6 shows that the association between female labor and health outcomes remain robust to the inclusion of the presence of large animals and dependence on animal husbandry as covariates in the model. Table S7 present the results of additional covariates added to the model one by one.

Table S8 shows that the association between ancestral arable land and female health outcomes remain significant despite accounting for female labor force participation rate. Table S9 presents the results of all agro-ecological factors, supporting the discussion in the last section of the paper.

Table S1: Cross-correlation table

Variables	Migration-adjusted potential arable land	Ancestral arable land	Years since neolithic transition	Ancestral plough use	Pre-1500 CE crop yield	Log(per capita income)	Land area in the tropics	Distance from nearest coastline/ river
Migration-adjusted potential arable land	1.00							
Ancestral arable land	0.44*** (<0.01)	1.00						
Years since neolithic transition	-0.32*** (<0.01)	-0.22** (0.01)	1.00					
Ancestral plough use	-0.23*** (0.01)	0.15* (0.09)	0.55*** (<0.01)	1.00				
Pre-1500 CE crop yield	0.52*** (<0.01)	0.31*** (<0.01)	0.10 (0.26)	0.06 (0.53)	1.00			
Log(per capita income)	-0.05 (0.54)	0.10 (0.27)	0.44*** (<0.01)	0.47*** (<0.01)	0.19** (0.03)	1.00		
Land area in in the tropics	0.22** (0.01)	-0.33*** (<0.01)	-0.55*** (<0.01)	-0.74*** (<0.01)	-0.17* (0.06)	-0.58*** (<0.01)	1.00	
Distance from nearest coastline/river	-0.29*** (<0.01)	0.04 (0.66)	-0.13 (0.14)	-0.20** (0.02)	-0.40*** (<0.01)	-0.31*** (<0.01)	0.02 (0.85)	1.00

$p-$values in parentheses. * $p < 0.10$, ** $p < 0.05$, *** $p < 0.01$.

Table S2: Correlation between historical factors

Variables	Migration-adjusted potential arable land	Ancestral Arable Land	Migration-adjusted years since neolithic transition	Ancestral plough use	Ancestry-adjusted Pre-1500 CE average crop yield	Plough positive crops	Plough negative crops
Migration-adjusted potential arable land	1.00						
Ancestral arable land	0.444*** (0.00)	1.00					
Migration-adjusted years since neolithic transition	-0.320*** (<0.01)	-0.222** (0.010)	1.00				
Ancestral plough use	-0.228*** (0.008)	0.149* (0.086)	0.55*** (<0.01)	1.00			
Ancestry-adjusted Pre-1500 CE average crop yield	0.520*** (<0.01)	0.313*** (<0.01)	0.982 (0.261)	0.055 (0.526)	1.00		
Plough positive crops	-0.192** (0.028)	0.363*** (<0.01)	0.559*** (<0.01)	0.773*** (<0.01)	0.194** (0.026)	1.00	
Plough negative crops	0.273*** (<0.01)	0.005 (0.954)	-0.572*** (<0.01)	-0.591*** (<0.01)	0.035 (0.688)	-0.703*** (<0.010)	1.00

$p-$values in parentheses. * $p < 0.10$, ** $p < 0.05$, *** $p < 0.01$.

Table S3: Correlation between gender outcomes in 2012

Variables	Female Labor Force Participation	Maternal Mortality Ratio	Female-Male Life Expectancy Gap	Share of Women in National Parliaments	Female-Male Secondary Education Gap
Female Labor Force Participation	1.00				
Maternal Mortality Ratio	0.327*** (<0.01)	1.00			
Female-Male Life Expectancy Gap	-0.106 (0.221)	-0.554*** (<0.01)	1.00		
Share of Women in National Parliaments	0.224*** (<0.01)	-0.042 (0.631)	-0.002 (0.983)	1.00	
Female-Male Secondary Education Gap	0.061 (0.481)	-0.341*** (<0.01)	0.201** (0.020)	0.17* (0.053)	1.00

$p-$values in parentheses. * $p < 0.10$, ** $p < 0.05$, *** $p < 0.01$.

Table S4: Ancestral Arable Land and Female Labor Force Participation without GDP per Capita

	(1)	(2)	(3)	(4)
Ancestral arable land	19.78***	19.74***	13.99***	10.92**
	(5.161)	(5.009)	(4.541)	(4.401)
Land area in the geographical tropics	13.62***	12.10**	5.160	6.633
	(4.643)	(5.432)	(5.227)	(4.979)
Distance from nearest coastline/river	3.509	2.871	2.285	5.335**
	(2.415)	(2.663)	(2.654)	(2.563)
Ancestral plough use		-4.036	-4.810	-3.302
		(6.251)	(6.290)	(5.469)
Years since neolithic transition			-4.413***	-4.507***
			(0.973)	(0.997)
Pre-1500 CE crop yield				0.00606***
				(0.00202)
Continent Dummies	Yes	Yes	Yes	Yes
Observations	134	134	133	133
Adjusted R^2	0.359	0.358	0.464	0.499

The dependent variable is female labor force participation rate. Each column presents the results of a different multivariate regression specification. The significance of each explanatory variable is assessed using a $t-$test. OLS coefficients are reported with robust standard errors in parentheses. * $p < 0.10$, ** $p < 0.05$, *** $p < 0.01$. Constant not reported.

Table S5: Ancestral Arable Land and Female Labor Force Participation: Robustness without GDP per Capita

	(1)	(2)	(3)	(4)	(5)	(6)	(7)
Ancestral arable land	20.53***	20.31***	18.05***	17.74***	17.64***	12.85***	11.10**
	(4.777)	(4.679)	(4.149)	(4.190)	(4.142)	(4.301)	(4.550)
Ancestral plough use						-2.336	-1.523
						(5.200)	(4.633)
Years since neolithic transition						-3.673***	-3.720***
						(1.089)	(1.134)
Pre-1500 CE crop yield							0.00615***
							(0.00222)
Democracy Index (2000)	0.131	0.0823	0.176	0.294	0.259	0.0886	-0.0159
	(0.318)	(0.327)	(0.313)	(0.324)	(0.360)	(0.352)	(0.335)
Legal origin: France		-0.168	1.811	4.043	3.853	3.978	1.865
		(3.589)	(3.678)	(3.809)	(3.814)	(3.557)	(3.436)
Legal origin: Germany		4.203	3.652	5.196	5.551	3.381	1.028
		(3.719)	(3.777)	(4.164)	(4.106)	(4.174)	(4.534)
Legal origin: Scandinavia		10.01***	14.49***	16.81***	17.61***	11.64**	15.53***
		(3.712)	(4.432)	(4.659)	(4.586)	(5.322)	(5.668)
Religious Fractionalization			17.05**	20.35***	20.80***	20.69***	16.91***
			(6.982)	(6.768)	(6.592)	(6.434)	(6.300)
State Antiquity Index				-0.978	-0.301	4.166	2.921
				(7.680)	(8.167)	(7.852)	(8.055)
Share of agriculture in GDP					14.859	14.39	10.24
					(18.559)	(16.01)	(14.93)
Share of industry in GDP					-5.724	-11.63	-11.28
					(14.667)	(13.72)	(12.39)
Baseline controls	Yes	Yes	Yes	Yes	Yes	Yes	Yes
Continent dummies	Yes	Yes	Yes	Yes	Yes	Yes	Yes
Observations	130	130	130	122	122	122	122
Adjusted R^2	0.364	0.362	0.397	0.418	0.423	0.484	0.511

The dependent variable is female labor force participation rate. Each column presents the results of a different multivariate regression specification. The significance of each explanatory variable is assessed using a $t-$test. OLS coefficients are reported with robust standard errors in parentheses. * $p < 0.10$, ** $p < 0.05$, *** $p < 0.01$. Omitted legal origin: British. Baseline controls: Log(per capita income) and its squared term, land area in geographical tropics, distance to nearest coastline or sea-navigable river. Constant not reported.

Table S6: Ancestral Arable Land and Female Labor and Health Outcomes (Controlling for Large Animals and Husbandry)

	Female Labor Force Participation		Maternal Mortality Ratio		Female-Male Life Expectancy Gap	
	(1)	(2)	(3)	(4)	(5)	(6)
Ancestral arable land	9.736**	12.11**	-137.9***	-114.5**	1.576**	1.813**
	(4.639)	(5.249)	(46.79)	(55.68)	(0.654)	(0.715)
Ancestral plough use	-6.390	-2.552	37.95	61.25*	-0.650	-0.308
	(4.404)	(4.297)	(30.94)	(34.02)	(0.676)	(0.712)
Years since neolithic transition	-4.386***	-3.802***	-1.112	-4.028	-0.198	-0.216
	(1.018)	(1.200)	(6.365)	(7.337)	(0.135)	(0.145)
Pre-1500 CE crop yield	0.0067***	0.0073***	-0.0154	-0.0151	0.0009***	0.0006**
	(0.0022)	(0.0025)	(0.0120)	(0.0164)	(0.0003)	(0.0003)
Presence of large animals	14.47***	13.40***	58.42	37.27	-0.398	-0.470
	(3.790)	(4.664)	(36.11)	(38.96)	(1.034)	(1.043)
Dependence on animal husbandry for subsistence	-23.50**	-16.87	-54.46	-11.89	0.645	1.719
	(10.03)	(10.85)	(65.31)	(65.35)	(1.472)	(1.492)
Baseline controls	Yes	Yes	Yes	Yes	Yes	Yes
Additional controls		Yes		Yes		Yes
Continent dummies	Yes	Yes	Yes	Yes	Yes	Yes
Observations	132	121	132	121	132	121
Adjusted R^2	0.560	0.542	0.790	0.797	0.624	0.669

The dependent variable is female labor force participation rate in columns 1–2, maternal mortality ratio in columns 3–4, and female-male life expectancy gap in columns 5–6. Each column presents the results of a different multivariate regression specification. The significance of each explanatory variable is assessed using a $t-$test. OLS coefficients are reported with robust standard errors in parentheses. * $p < 0.10$, ** $p < 0.05$, *** $p < 0.01$. Baseline controls: Log(per capita income) and its squared term, land area in geographical tropics, distance to nearest coastline or sea-navigable river. Additional controls: democracy index, legal origin dummies, state antiquity index, religious fractionalization, shares of agriculture and industry in GDP. Constant not reported.

Table S7: Ancestral Arable Land and Female Labor Force Participation: Robustness

	(1)	(2)	(3)	(4)	(5)	(6)	(7)
Ancestral arable land	19.25***	18.57***	15.60***	16.00***	17.74***	13.38***	12.02**
	(5.411)	(5.395)	(4.824)	(4.748)	(4.920)	(4.979)	(5.221)
Ancestral plough use						-1.316	0.0667
						(5.292)	(4.657)
Years since neolithic transition						-3.745***	-3.844***
						(1.087)	(1.157)
Pre-1500 CE crop yield							0.00730***
							(0.00235)
Democracy Index (2000)	0.0830	0.0377	0.145	0.214	0.159	-0.0460	-0.230
	(0.322)	(0.326)	(0.309)	(0.364)	(0.388)	(0.371)	(0.355)
Legal origin: France		-0.535	1.374	3.859	4.188	4.544	2.354
		(3.592)	(3.642)	(3.853)	(3.882)	(3.592)	(3.397)
Legal origin: Germany		4.912	4.327	6.137	6.364	4.304	1.806
		(3.740)	(3.813)	(4.177)	(4.201)	(4.295)	(4.676)
Legal origin: Scandinavia		6.737*	11.93***	13.94***	13.64***	7.210	10.38*
		(3.677)	(4.313)	(4.611)	(4.785)	(5.839)	(5.959)
Religious Fractionalization			18.22***	19.97***	19.27***	18.90***	13.60**
			(6.665)	(6.672)	(6.741)	(6.678)	(6.584)
State Antiquity Index				-4.539	-4.346	-0.257	-3.007
				(8.575)	(8.937)	(8.707)	(9.079)
Share of agriculture in GDP					17.03	19.69	19.76
					(20.509)	(18.65)	(17.38)
Share of industry in GDP					-2.160	-8.542	-8.065
					(14.934)	(13.67)	(12.09)
Observations	130	130	130	122	122	122	122
Adjusted R^2	0.385	0.381	0.422	0.432	0.427	0.492	0.531
Baseline controls	Yes	Yes	Yes	Yes	Yes	Yes	Yes
Continent dummies	Yes	Yes	Yes	Yes	Yes	Yes	Yes

The dependent variable is female labor force participation rate. Each column presents the results of a different multivariate regression specification. The significance of each explanatory variable is assessed using a $t-$test. OLS coefficients are reported with robust standard errors in parentheses. * $p < 0.10$, ** $p < 0.05$, *** $p < 0.01$. Omitted legal origin: British. Baseline controls: Log(per capita income) and its squared term, land area in geographical tropics, distance to nearest coastline or sea-navigable river. Constant not reported.

Table S8: Ancestral Arable Land and Women's Health Controlling for Current Female Labor Force Participation

	Maternal Mortality Ratio				Female-Male Life Expectancy Gap			
	(1)	(2)	(3)	(4)	(5)	(6)	(7)	(8)
Ancestral arable land	-122.7**	-127.1***	-127.3**	-88.97#	1.183*	1.789***	1.505**	1.778**
	(50.66)	(48.32)	(49.06)	(55.68)	(0.688)	(0.577)	(0.611)	(0.690)
Ancestral plough use			46.01	65.49**			-0.740	-0.458
			(28.16)	(32.47)			(0.635)	(0.677)
Years since neolithic transition			-4.376	-10.18			-0.211	-0.216
			(6.566)	(7.105)			(0.166)	(0.169)
Pre-1500 CE crop yield			-0.0124	-0.0064			0.0009***	0.0006*
			(0.0119)	(0.0148)			(0.0003)	(0.00035)
Female Labor Force Participation	0.522	-0.694	-0.661	-1.601	0.0093	0.0157	-0.004	-0.0017
	(1.005)	(0.804)	(0.935)	(0.988)	(0.0144)	(0.0128)	(0.0166)	(0.0160)
Baseline controls		Yes	Yes	Yes		Yes	Yes	Yes
Additional controls				Yes				Yes
Continent dummies	Yes	Yes	Yes	Yes	Yes	Yes	Yes	Yes
Observations	134	134	133	122	134	134	133	122
Adjusted R^2	0.688	0.795	0.794	0.807	0.361	0.590	0.620	0.661

The dependent variable is maternal mortality ratio in columns 1–4 and female-male life expectancy gap in columns 5–8. Each column presents the results of a different multivariate regression specification. The significance of each explanatory variable is assessed using a $t-$test. OLS coefficients are reported with robust standard errors in parentheses. $^{\#}$ $p = 0.113$, * $p < 0.10$, ** $p < 0.05$, *** $p < 0.01$. Baseline controls: Log(per capita income) and its squared term, land area in geographical tropics, distance to nearest coastline or sea-navigable river. Additional controls: democracy index, legal origin dummies, state antiquity index, religious fractionalization, shares of agriculture and industry in GDP. Constant not reported.

Table S9: Horse Race Among Historical Agro-Ecological Factors

	Female Labor Labor Force Participation	Maternal Mortality Ratio	Female-Male Life Expectancy Gap	Female-Male Secondary Education gap	Share of Women in Parliament
	(1)	(2)	(3)	(4)	(5)
Ancestral arable land	9.913***	-117.2**	1.298*	-1.104	4.414
	(3.766)	(50.15)	(0.728)	(3.563)	(3.695)
	[0.195]	[-0.175]	[0.163]	[-0.042]	[0.130]
Ancestral plough use	-7.805	-5.131	-1.720**	2.565	0.133
	(5.553)	(34.52)	(0.749)	(2.450)	(2.907)
	[-0.225]	[-0.011]	[-0.316]	[0.144]	[0.006]
Years since neolithic transition	-4.885***	-6.589	-0.211	-1.144**	-0.772
	(0.993)	(7.487)	(0.144)	(0.498)	(0.627)
	[-0.622]	[-0.064]	[-0.172]	[-0.283]	[-0.147]
Pre-1500 CE crop yield	0.00460**	-0.00177	0.000429	-0.00195*	-0.000961
	(0.00192)	(0.0142)	(0.000352)	(0.00110)	(0.00166)
	[0.197]	[-0.006]	[0.117]	[-0.162]	[-0.062]
Continent Dummies	Yes	Yes	Yes	Yes	Yes
Observations	133	133	133	133	133
Adjusted R^2	0.486	0.683	0.403	0.120	0.074

The dependent variable is maternal mortality ratio in columns 1–4 and female-male life expectancy gap in columns 5–8. Each column presents the results of a different multivariate regression specification. The significance of each explanatory variable is assessed using a $t-$test. OLS coefficients are reported with robust standard errors in parentheses. * $p < 0.10$, ** $p < 0.05$, *** $p < 0.01$. Constant not reported.

Validity of the Measures of Historical Arable Land

If the observed association between ancestral arable land and contemporary female labor force participation were driven by the historical influence on culture, then current potential arable land should be correlated with the female labor force participation in the Old World but not in the New World. After all, female labor force participation in the New World, extensively repopulated after 1492 CE, ought to derive in large part from the cultures of its European, African, and Asian settlers, influenced by conditions in their Old World nations of origin. On the other hand, since it takes this re-population into account, the correlation between ancestral arable land and female labor force participation should be significant for both the New World and the Old World. This can be tested by including our measures of historical availability of arable land interacted with an indicator of nations in the Americas and Oceania in the baseline specification.

The resulting estimates, presented in Table S10, indicate that whereas current potential arable land is negatively statistically significantly correlated with the female labor force participation in the Old World, correlation between these variables is not statistically significantly different from zero in the New World. An F-test fails to reject the null hypothesis that the coefficients of the *current potential arable land* and *current potential arable land* $\times$ *Dummy for countries in the New World* sum to zero. On the other hand, the negative correlation between ancestral arable land and female labor force participation is statistically significant at conventional levels for both the New World and the Old World. Not only is the coefficient of the interaction term between ancestral arable land and the dummy for countries in the New World is much smaller and statistically insignificant, but the linear combination of ancestral arable land and the interaction term is positive and statistically significant. These results point to the historical influence of culture, and are consistent with our claim that the observed effects are due to the transmission of norms.

Table S10: Historicity of Ancestral Arable Land

	(1)	(2)
**Panel A**		
Current potential arable land	22.55***	
	(7.312)	
Current potential arable land × New World countries	-22.44**	
	(10.11)	
New World countries	41.55***	
	(6.385)	
Current potential arable land +	0.105	
(Current potential arable land × New World countries)	(7.895)	
H_0: Current potential arable land + (Current potential arable land × New World countries) = 0 ⇒ $p = 0.9894$		
**Panel B**		
Ancestral arable land		20.123***
		(6.642)
Ancestral arable land × New World countries		-7.530
		(7.382)
New World countries		39.215***
		(7.462)
Ancestral arable land +		12.593**
(Ancestral arable land × New World countries)		(5.459)
H_0: Ancestral arable land + (Ancestral arable land × New World countries) = 0 ⇒ $p = 0.0228$		
Baseline controls	Yes	Yes
Continent dummies	Yes	Yes
Observations	133	134
Adjusted R^2	0.385	0.382

The dependent variable is female labor force participation rate. Each column presents the results of a different multivariate regression specification. The significance of each explanatory variable is assessed using a $t-$test. OLS coefficients are reported with robust standard errors in parentheses. * $p < 0.10$, ** $p < 0.05$, *** $p < 0.01$. Baseline controls: Log(per capita income) and its squared term, land area in geographical tropics, distance to nearest coastline or sea-navigable river. Constant not reported.

Ancestral vs. Current Arable Land: The Role of Norms in Female Health Outcomes

In Table S11, we present the results of a horse race between ancestral and current potential arable land for women's health and empowerment indicators. Consistent with our hypotheses, we find that ancestral arable land is negatively, significantly associated with the maternal mortality ratio and positively, significantly correlated with female-male life expectancy gap. The current potential arable land, on the other hand, is significantly associated with only female-male life expectancy gap. Overall, these results support our hypothesis that the availability of ancestral arable land continues to impact women's health outcomes even today through the persistence of cultural norms. Importantly, these results suggest that availability of land plays a more important role through shaping gender norms than influencing current resource environments.

Table S11: Women Health and Empowerment: A Horse Race between Ancestral vs. Current Arable Land

	Maternal Mortality Ratio		Female-Male Life Expectancy Gap		Female-Male Secondary Education Gap		Share of Women in Parliament	
	(1)	(2)	(3)	(4)	(5)	(6)	(7)	(8)
Ancestral arable land		-145.0***		1.227**		-0.328		4.111
		(54.67)		(0.569)		(3.804)		(4.501)
Current potential arable land	-51.75	14.36	2.921***	2.362***	-1.036	-0.886	-3.198	-5.073
	(40.18)	(50.77)	(0.658)	(0.676)	(3.234)	(3.821)	(4.285)	(4.828)
Baseline Controls	Yes	Yes	Yes	Yes	Yes	Yes	Yes	Yes
Continent dummies	Yes	Yes	Yes	Yes	Yes	Yes	Yes	Yes
Observations	133	133	133	133	133	133	133	133
Adjusted R^2	0.773	0.792	0.604	0.611	0.244	0.238	0.097	0.096

The dependent variable is maternal mortality ratio in columns 1–2, female-male life expectancy gap in columns 3–4, female-male secondary education gap in columns 5–6, and the share of women in national parliaments in columns 7–8. Each column presents the results of a different multivariate regression specification. The significance of each explanatory variable is assessed using a $t-$test. OLS coefficients are reported with robust standard errors in parentheses. * $p < 0.10$, ** $p < 0.05$, *** $p < 0.01$. Baseline controls: Log(per capita income) and its squared term, land area in geographical tropics, distance to nearest coastline or sea-navigable river. Constant not reported.
